# Nanoscale Surface-Enhanced Raman Spectroscopy Investigation of a Polyphenol-Based Plasmonic Nanovector

**DOI:** 10.3390/nano13030377

**Published:** 2023-01-17

**Authors:** Giacomo Nisini, Annalisa Scroccarello, Francesca Ripanti, Claudia Fasolato, Francesco Cappelluti, Angela Capocefalo, Flavio Della Pelle, Dario Compagnone, Paolo Postorino

**Affiliations:** 1 Physics Department, Sapienza University, P.le A. Moro 5, 00185 Rome, Italy; 2 Department of Bioscience and Technology for Food, Agriculture and Environment, University of Teramo, via R. Balzarini 1, 64100 Teramo, Italy; 3 Physics and Geology Department, University of Perugia, via A. Pascoli, 06123 Perugia, Italy; 4 Institute for Complex Systems, National Research Council, P.le A. Moro 5, 00185 Rome, Italy; 5 Teknologisk Institut, Kongsvang Allé 29, 8000 Aarhus, Denmark

**Keywords:** functional nanoparticles, bioplasmonic nanomaterial, SERS spectroscopy, polyphenol, nanovector, green synthesis of nanomaterials

## Abstract

The demand for next-generation multifunctional nanovectors, combining therapeutic effects with specific cellular targeting, has significantly grown during the last few years, pursuing less invasive therapy strategies. Polyphenol-conjugated silver nanoparticles (AgNPs) appear as potential multifunctional nanovectors, integrating the biorecognition capability and the antioxidant power of polyphenols, the antimicrobial activity of silver, and the drug delivery capability of NPs. We present a spectroscopic and microscopic investigation on polyphenol-synthesized AgNPs, selecting caffeic acid (CA) and catechol (CT) as model polyphenols and using them as reducing agents for the AgNP green synthesis, both in the presence and in the absence of a capping agent. We exploit the plasmonic properties of AgNPs to collect Surface-Enhanced Raman Scattering (SERS) spectra from the nanosized region next to the Ag surface and to characterize the molecular environment in the proximity of the NP, assessing the orientation and tunable deprotonation level of CA, depending on the synthesis conditions. Our results suggest that the SERS investigation of such nanovectors can provide crucial information for their perspective biomedical application.

## 1. Introduction

Polyphenols are a wide class of chemical compounds common in several living species, sharing the phenol elementary unit, consisting of a phenyl group bonded to a hydroxyl group. They are considered great inhibitors of oxidative stress, capable of hindering the biological damages of free radicals and reactive oxygen species [1,2], thanks to the antioxidant properties mainly associated with the hydroxyl groups [3,4]. The effects of polyphenol assumption on health have been widely demonstrated [5,6,7,8]. Even more importantly, polyphenols show a strong binding affinity toward different biomolecules, including nucleic acids, proteins, and phospholipids [9,10], which makes them strong candidates for efficient drug delivery strategies, targeting specific receptors or biomolecules [11,12].

Nanovectors, i.e., 1–100 nm sized artificial materials, have emerged as a powerful tool to transport drugs in a controlled and targeted way. Possible nanovectors are liposomes, phospholipid complexes, niosomes, protein-based nanoparticles (NPs), micelles, emulsions, and noble metal (Ag, Au) NPs [13], which could be exploited for different applications [14,15,16,17,18]. Remarkably, AgNPs can be synthesized directly using polyphenols as reducing agents [19]: this is a scalable, biocompatible approach avoiding the conventional toxic reagents [20]. Such a strategy allows obtaining a high load of polyphenol molecules on the NP surface, constructing an efficient biointerface while avoiding a posteriori functionalization. This green synthesis of AgNPs can be realized using different polyphenol species [21,22]. Recent studies on the in vitro nanotoxicity of polyphenol-AgNPs demonstrated that different phenolic compounds, depending on their chemistry, structure, and antioxidant capacity, modulate the antimicrobial effect and the mechanism of action of the nanovectors [21,23,24]. Remarkably, it is observed that polyphenol-synthesized AgNP nanovectors couple the beneficial effects and specific targeting capability of polyphenols to the antibacterial activity of AgNPs, thus representing a multifunctional nanomaterial suitable for nanomedicine and nanobiotechnology applications [23,25,26,27,28]. Furthermore, recent investigations on polyphenol-conjugated AgNPs have reported a reduced or absent toxicity versus human keratinocytes [19] and even a selective toxicity against cancer cells compared to normal ones [29,30].

However, one of the main problems related to pharmaceutical applications of such composite systems is the lack of knowledge of the specific chemical composition of the nanomaterial surface, which indeed mainly determines its interacting properties and, if not properly controlled, guide to side-effects and non-desired behaviors of the system. By exploiting the significant signal enhancement (typically, 6-8 orders of magnitude) due to the localized surface plasmon resonance (LSPR) of noble metal nanostructures and the molecular specificity of Raman spectroscopy, Surface-Enhanced Raman Scattering (SERS) spectroscopy can be an extraordinary tool to access the real chemical state of molecules in the proximity of metal NPs, thus providing important information on the surface chemical properties of the system [31,32]. In the perspective of exploiting polyphenol-AgNPs as new multifunctional nanovectors, the use of an ultrasensitive and molecular-specific probe can be crucial for a thorough investigation of the system beyond the extent of conventional analytical methods. This is a necessary step to assay the properties and potentialities of such nanovectors toward biomedical applications.

In the present work, caffeic acid (CA)-synthesized AgNPs were investigated as a case study, both in the presence and in the absence of the cetyltrimethylammonium chloride capping agent (CPP). Catechol (CT) was selected as a reference system since the CT moiety is a molecular subunit of CA. A systematic analysis of the Raman and SERS response of CA and CT polyphenols as a function of pH, obtained using standard Ag colloids as the SERS substrate, along with the comparison of experimental spectra to Density Functional Theory (DFT) calculations, was used as a reference for the spectroscopic investigation of the polyphenol-AgNP nanovectors under examination. SERS nano-spectroscopy allowed not only the detection of the CA layer at the AgNP surface, confirming its stability after the synthesis reaction, but also the determination of the molecular orientation at the metal interface and, thus, of the functional groups exposed on the nanomaterial surface. Furthermore, we demonstrated that the level of deprotonation of the CA molecules can be tuned depending on the synthesis conditions, opening interesting biomedical application perspectives.

## 2. Materials and Methods

All the chemicals were of analytical grade. CA and CT powders were purchased from Merck KGaA (Darmstadt, Germany) and Extrasynthese (Genay, France), respectively. Cetyltrimethylammonium chloride (25% in water), silver nitrate (AgNO_3_, > 99%), sodium hydroxide (NaOH), and hydrochloric acid (HCl) were purchased from Merck KGaA and used without further purification.

### 2.1. CA and CT Solutions for Raman Measurements

Water solutions of 10 mM CA and 1 M CT were prepared for Raman measurements as a function of pH. Tiny amounts of highly concentrated HCl and NaOH solutions were added to the polyphenol solutions until reaching the desired pH values without significantly altering the polyphenol concentrations. In the CA case, measurements were collected, keeping the sample at 60 °C to increase its solubility (∼5.6 mM at room temperature) [33]. CA and CT methanol solutions were prepared at 0.1 and 2 M, respectively (Section A of Supplementary Materials).

### 2.2. Polyphenol-Synthesized AgNPs

Polyphenol-induced AgNP synthesis was carried out both in the presence and in the absence of the CPP. The synthesis of CA-functionalized AgNPs in the presence of CPP (CPP-CA@AgNPs) was performed according to [21]. In brief, the reaction took place at a final volume of 1 mL. A total of 10 µL of 0.8 mM CPP, 50 µL of 5 mM AgNO_3_, 40 µL of 1 mM CA, and 20 µL of 5 M NaOH were added to 880 µL of ultrapure (milli-Q) deionized water. Final concentrations of 8 µM CPP, 250 µM AgNO_3_, and 40 µM CA were reached, with a final pH of 13. The reaction mix was stirred for 10 min in an orbital shaker at 300 rpm, and then the reaction was blocked in ice for 10 min. For the CA-synthesized AgNPs without CPP (CA@AgNPs), the reaction was performed according to [23] with minor modifications. The reaction took place at a final volume of 10 mL. A total of 250 µL of 40 mM AgNO_3_, 125 µL of 40 mM CA, and 100 µL of 1 M NaOH was added to 9.525 mL milli-Q water. The reaction mix was stirred for 30 s. The final concentrations of 1 mM AgNO_3_ and 500 µM CA were reached, with a final pH of 12. Analogous procedures were carried out to prepare CT-synthesized AgNPs with (CPP-CT@AgNPs) and without (CT@AgNPs) CPP. After NP synthesis, all the samples were purified by centrifugation and resuspended in water at the aforementioned pH conditions. The prepared AgNPs were promptly used within 10 weeks, according to previous stability studies [23].

### 2.3. Hydroxylamine-Synthesized AgNPs

Reference AgNPs were prepared according to the well-established Leopold method [34], employing hydroxylammonium chloride as the reducing agent for Ag ions in the solution. A total of 17 mg of AgNO_3_ powder was dissolved in 90 mL of milli-Q water. Simultaneously, 21 mg of hydroxylammonium chloride and 5 mL of 0.1 M NaOH were added to 5 mL of water. The solutions were mixed and stirred for 20 min. The synthesized NPs were characterized by SEM, UV-Vis absorption, and Raman spectroscopy. The polyphenol conjugation of hydroxylamine-synthesized AgNP (Hyd@AgNP) was induced by mixing 200 µL AgNP solution with 20 µL of polyphenol water solution (4 mM CA, 1 M CT) and 20 µL of 10 mM magnesium sulfate (MgSO_4_) solution. Similarly, the CPP SERS reference spectrum was also acquired from Hyd@AgNPs, mixed with 0.8 mM CPP water solution.

### 2.4. SEM Images

SEM images were collected in backscattering mode with an AURIGA-Zeiss model HR-FESEM platform at the Sapienza Nanotechnology and Nanoscience Laboratory. Images were acquired with different magnifications. Extra High Tension was kept fixed at 30 kV. For each analyzed sample, ∼5 µL of AgNP dispersion was dropcasted on a TEM grid. Measurements were collected on dried samples.

### 2.5. Dynamic Light Scattering, ζ-Potential and Light Transmission Spectroscopy Measurements

Dynamic light scattering and ζ-potential measurements were performed by employing a NanoZetaSizer apparatus (Malvern Instruments LTD) equipped with a 5 mW He-Ne laser. Details on the dynamic light scattering analysis are presented in Section E of the Supplementary Materials. ζ-potential values were obtained by combining laser Doppler velocimetry and phase analysis light scattering to determine the average electrophoretic mobility $\mu_{e}$. The measured values were then converted into ζ-potential using the Smoluchowski relation: $\zeta =\frac{\mu_{e}\eta }{\epsilon }$; where η and ϵ are the solvent viscosity and permittivity, respectively.

The particle size distribution of the different AgNPs samples was obtained by light transmission spectroscopy (LTS). The optical extinction spectrum of the samples was measured in a wide wavelength range, between 190 and 1300 nm, by employing a double ray spectrophotometer (v-590, Jasco) and quartz cuvettes with 1 mm path length. The data analysis procedure, based on Mie scattering, is discussed in reference [35].

### 2.6. Raman and SERS Spectroscopy

Raman and SERS measurements were performed with a Horiba HR-Evolution microspectrometer in backscattering geometry. The spectrometer is equipped with a He-Ne laser (λ = 632.8 nm). The laser power at the sample surface, ∼10 mW, can be attenuated with a set of neutral density filters. A 600 lines/mm diffraction grating ensures a spectral resolution better than 3 cm^−1^. The detector is a Peltier-cooled charge-coupled device (CCD). The spectrometer is coupled with a confocal microscope, and a set of objectives with different magnification is available. In this experiment, the 50× objective (numerical aperture 0.5, laser spot diameter 1.5 µm) was selected for Raman measurements on liquid and powder samples, while the 100× (numerical aperture 0.8, laser spot diameter 1 µm) was selected for Raman and SERS measurements on dried samples.

The Raman spectra of polyphenols at the solid state were collected after the deposition of some powder grains on a glass slide. Raman spectra in solution were obtained using a quartz cuvette filled with 0.1 mL of polyphenol solution. Spectral contributions from the solvents were measured and subtracted during the data analysis process. SERS measurements were performed on dried samples to allow for the NP packing into aggregates, where the so-called hotspots, i.e., the small gaps between close-packed NPs majorly contributing to the SERS field enhancement, are frequent and maximize the signal amplification [36,37]. The SERS spectra shown here are obtained by averaging several spectra acquired from different NP aggregates on the sample. To avoid sample degradation, Raman and SERS spectra were collected using a laser power of about 10 mW for 10 s and 100 µW for 300 s, respectively.

### 2.7. Density Functional Theory Calculations

Calculations were performed using Gaussian 16 (rev. C.01) [38] on Galileo HPC from Cineca. Geometrical optimizations using the default convergence criteria were followed by analytical harmonic frequency calculations. No imaginary frequencies were found, therefore, testifying that true energy minima were attained. Since the chemical enhancement (CE) phenomenon of SERS is strongly correlated with the charge-transfer processes between the metal NPs and the probe molecule [39], and since the commonly employed hybrid functionals are known to qualitatively fail in dealing with this phenomenon [40], we choose to employ a long-range corrected DFT functional, and we tested the three functionals of this family available in Gaussian 16, namely wB97X-D [41], LC-wHPBE [42], and CAM-B3LYP [43]. The empirical correction for dispersion GD3BJ [44] was added to the latter two. The former, on the contrary, includes, by default, the less-recent GD2 correction [45]. We performed the test of the different functionals on the CA in vacuum and compared the obtained Raman spectra with the experimental ones. The best matching was found for the wB97X-D functional, and we, therefore, choose to employ it for the rest of the study. We employed the aug-CC-pVTZ [46,47,48] basis set for all atoms except silver, which was described using the LANLDZ [49,50] basis set and the corresponding pseudo-potential [51]. The scaling factor of 0.957 was chosen for this level of theory [52]. As customarily performed in the literature [53,54,55], and having as grounded basis the “adatom” model [56], which states that the CE effect of SERS can be, with a good approximation, explained by the interaction with a single defective site on the NP surface, the SERS spectra were simulated for a system composed by the probe molecule and a single Ag atom. Many different coordination complexes were optimized, and the resulting SERS spectra are the weighted sums of their respective spectra (Section C of Supplementary Materials). The weighting was performed using the Boltzmann population distribution for the different complexes at a temperature of 298.15 K. As performed elsewhere [57], we choose to employ as Gibbs free energies those obtained by summing the single-point electronic energy calculated at the highly accurate B2PLYPD3 [44,58]/aug-CC-pVTZ//wB97X-D/aug-CC-pVTZ level of theory with the thermal correction to the free energy calculated at the wB97X-D/aug-CC-pVTZ level. The calculated Raman activities ($S_{i}$) were converted to the relative Raman intensities ($I_{i}$), i.e., quantities comparable to the experimental data, using the relationship [59,60,61]:

(1) $$I_{i}=\frac{{\left(\nu_{0}-\nu_{i}\right)}^{4}S_{i}}{\nu_{i}\left(1-e^{-h\nu_{i}c/kT}\right)}$$

where $\nu_{0}$ is the exciting laser wavenumber, $\nu_{i}$ is the wavenumber of the *i*-th vibrational mode, *h* is the Planck constant, *c* is the speed of light, *k* is the Boltzmann constant, and *T* is the temperature, used to calculate the Bose occupation of the initial state of the scattering process. The assignment of the theoretical normal modes was performed using the VMARD technique [62]. The Molden package [63] was employed for the inspection of the spectra. Concerning CA, considering its pK*_a_* value is lower than neutral pH, both its normal form and its deprotonated form, largely predominant at neutral conditions, were considered for calculations [64,65]. The calculations with the implicit solvent were performed using the IEFPCM method [66,67], as implemented in Gaussian 16.

## 3. Results and Discussion

### 3.1. pH-Response of Polyphenols by Raman Spectroscopy

**Figure 1:**
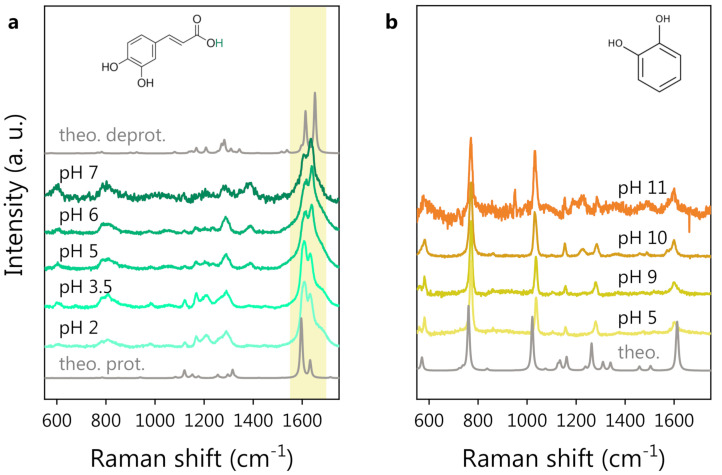
pH-dependent Raman study of polyphenols in water solution: (**a**) CA Raman spectra at different pH. The DFT spectra of protonated (bottom) and deprotonated CA (top, in the absence of the hydrogen atom colored green in the molecular structure) are also reported for reference in gray. The shadowed area highlights the major pH-induced spectral modifications. (**b**) CT Raman spectra at different pH. The CT molecular structure is shown at the top. The DFT spectrum of CT is also reported, in gray, at the bottom. Spectra are vertically shifted for clarity.

The experimental Raman spectra of CA and CT at different pH values are shown in Figure 1a,b, respectively. The DFT-calculated Raman spectra of CA and CT are also shown for reference. In the case of CA, both the protonated and deprotonated forms of the molecule were considered for DFT calculations since CA undergoes deprotonation within the explored pH range (pH ∼4.8).

**Figure 2:**
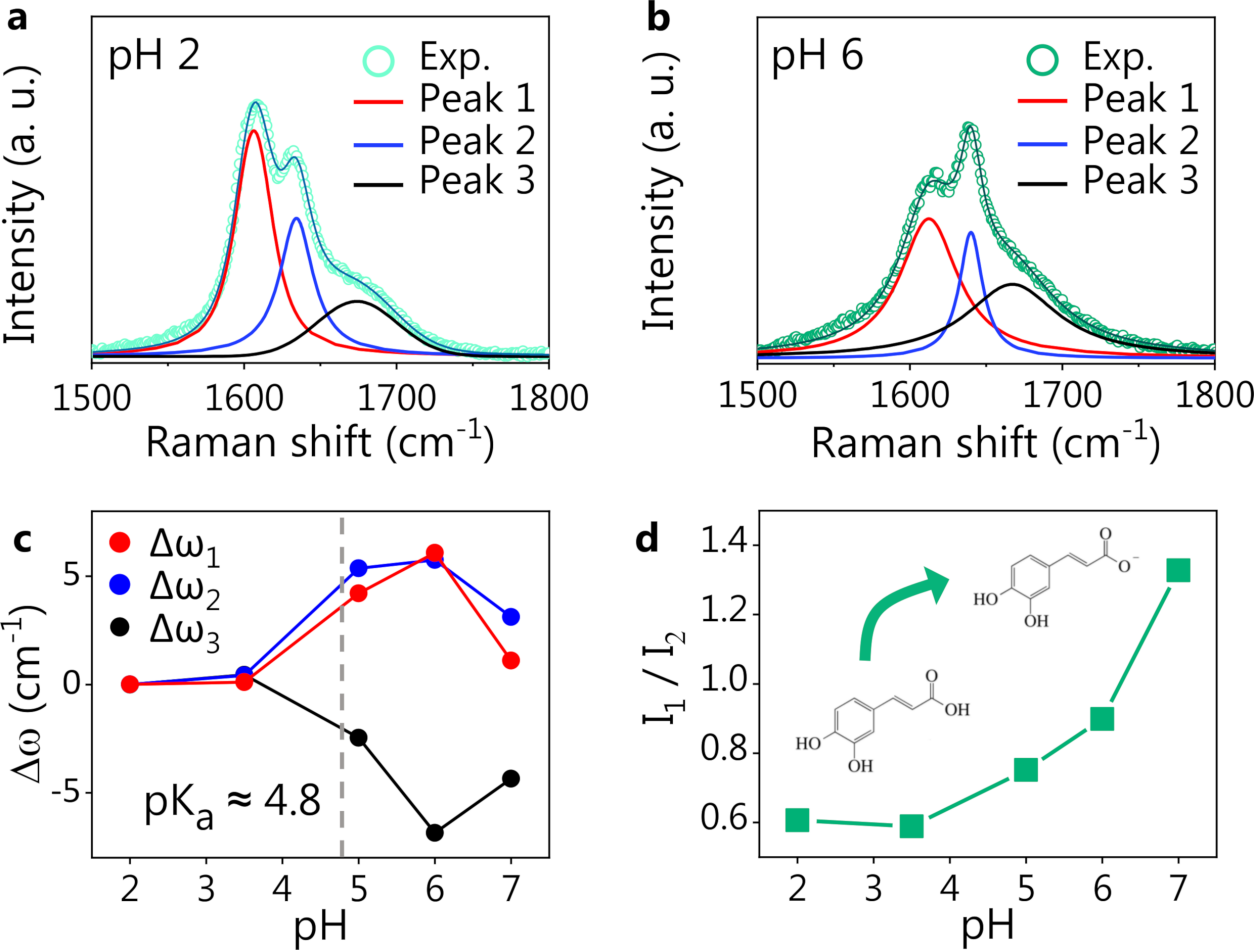
CA response to pH. Fitting deconvolution of the three most prominent peaks in the Raman spectrum of CA at pH 2 (**a**) and at pH 6 (**b**). The thick solid lines are the Voigt fitting components, and the thin solid line is the cumulative fitting, nicely reproducing the experimental data (dots). (**c**) Spectral shift of the peak position ($\Delta \omega =\omega_{pH}-\omega_{pH\;=\;2}$) for three fitting components as a function of pH. (**d**) Intensity ratio of the two strongest CA peaks (1 and 2) vs. pH.

We notice that the CT spectrum preserves its shape independently of the pH values, while the CA spectrum starts to change considerably around pH 5. Looking at Figure 1a, the most prominent spectral modification is a change in the shape of the structure around 1600 cm${}^{-1}$, with a spectral weight transfer from the band at 1607 cm${}^{-1}$ to that at 1632 cm${}^{-1}$ (experimental frequencies). We expect these spectral changes to be related to the deprotonation of the carboxylic group of CA. The hypothesis is supported by the comparison between the DFT-computed Raman spectra of protonated and deprotonated CA, where the same spectral modification can be noticed (see the shadowed area around 1600 cm${}^{-1}$). Bearing this effect in mind, one can state that a very good agreement between experimental and DFT-computed spectra for both CT and CA was observed. The DFT potential energy distribution analysis on protonated CA indeed suggests an assignment of the bands around 1600 cm^−1^ to the stretching vibrations of the C=C and C=O bonds. The deprotonation of the CA carboxylic group causes an electronic redistribution within the molecule, affecting the vibrational modes and, thus, accounting for the observed intensity and frequency changes in the Raman peaks. In Figure 2, we report a comparison between the CA spectrum at pH 2 (panel a) and pH 6 (panel b) in the 1500–1800 cm^−1^ spectral region. A three-component fitting deconvolution allows the monitoring of the changes in the peak position as a function of pH (Figure 2c). Δω is defined as the shift of each peak frequency taking pH 2 (Figure 2a) as reference, since it is associated with the fully protonated molecular form. Upon exceeding the nominal CA dissociation constant (pK_a_∼4.8), the three peak frequencies vary abruptly. As the pH further increases (>6), the band frequencies invert the previous trend, possibly suggesting the onset of a second deprotonation, in agreement with the literature, reporting a second pK_a_ at pH ∼8.6 [64,65]. Unfortunately, the CA Raman spectra at pH > 7 (data not shown) exhibited a strong fluorescence background, which did not allow to appreciate the CA Raman signature. The spectral weight transfer between the bands at 1607 and 1632 cm^−1^ upon CA deprotonation is quantifiable by the peak intensity ratio, shown in Figure 2d.

In the case of CT, even if the first deprotonation is expected at pH ∼9 [68,69], the Raman spectrum is not affected by any major variation in the pH range (5–11) explored. This is probably due to the small spectral weight associated with the hydroxyl group undergoing the deprotonation on the overall CT Raman signal. Indeed, the position and relative intensity of the Raman bands of CT is very well reproduced by the DFT-computed Raman spectrum of the protonated form of CT, shown at the bottom of Figure 1b. As in the case of CA, the fluorescence background prevented a spectroscopic investigation at higher pH values (>11). The CT spectrum at pH <5 (data not shown) was the same as that observed at pH 5, as reasonably expected.

In summary, while the CT Raman spectrum does not vary even at high pH, the spectrum of CA clearly reflects the modifications occurring in the molecule in correspondence with the characteristic dissociation conditions (pK*_a_*) (see Figure 1a and Figure 2). The pH response of CA can play a role in the development and application of the polyphenol-based nanovector, as will be discussed herein. Conveniently, CT can act as a reference molecule in the spectroscopic study of such nanovectors because of the structural similarity to CA and the fact that CT is not affected by pH variations. In the following section, we report on the acquisition of reference SERS spectra of CA and CT by employing a standard colloidal SERS substrate (Hyd@AgNPs) and on the SERS investigation, based on the reference measurements, of the novel CA-synthesized nanovectors (CA@AgNPs and CPP-CA@AgNPs).

### 3.2. Reference Polyphenol SERS Spectra

**Figure 3:**
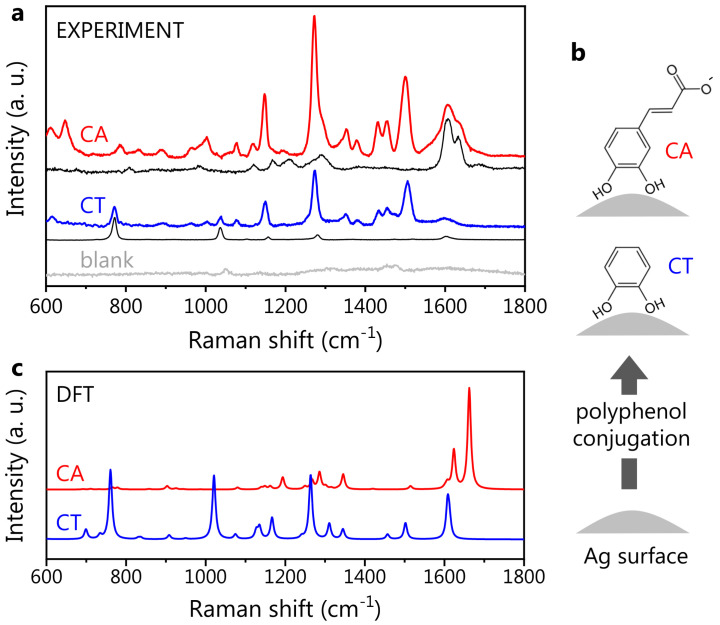
Polyphenol SERS signature. (**a**) Experimental SERS spectra of CA (red) and CT (blue), both obtained with Hyd@AgNPs as SERS substrate. The blank Hyd@AgNP spectrum is shown in light gray. Under each SERS spectrum, we reported the corresponding Raman one (black), collected in a water solution at neutral pH. For a description of data normalization, see the text. (**b**) Sketch showing the hypothesized molecular orientation for CA and CT, with the CT moiety adjacent to the AgNP surface. (**c**) Theoretical SERS spectra of CA (deprotonated form) and CT. These were calculated as a weighted average of different coordination complexes, i.e., interacting configurations between the molecule and a silver atom (see Section C of Supplementary Materials).

The SERS spectra of CA and CT, the latter mainly considered a model, being a molecular subunit of CA, were acquired as references for the polyphenol-nanovector characterization. The standard Hyd@AgNP colloid (see Materials and Methods for details) was employed as a SERS substrate. Indeed, the blank Hyd@AgNP spectrum is almost flat in the spectral region of interest: if one uses the characteristic low-frequency band assigned to Ag-related modes (at 240 cm^−1^) for the SERS spectra normalization [70,71,72], the spectral contribution of Hyd appears very weak: in Figure 3a, we show the spectra of CA- and CT-functionalized Hyd@AgNPs, along with the Raman spectra of CA and CT at neutral pH, and the blank SERS spectrum of the plasmonic substrate (Hyd@AgNPs). The polyphenol SERS spectra are normalized to the corresponding Raman ones via the most intense Raman peaks, for CT at ∼760 cm${}^{-1}$ and for CA at ∼1640 cm${}^{-1}$. For both CA and CT, the observed SERS spectral shapes are considerably different from the corresponding Raman ones. New vibrational peaks ascribed to the polyphenols appear in the 1100–1550 cm^−1^ spectral region.

In more detail, the difference observed between the Raman and SERS spectra is ascribed to the interaction between the polyphenols and the AgNP surface, inducing the activation of forbidden Raman modes: hence, this change is a marker of the effective polyphenol-NP conjugation [36]. The activated modes in CA and CT SERS spectra are very similar, suggesting not only their assignment to the common catechol unit (containing a phenolic ring, that is a moiety expectedly characterized by a strong SERS activity) but also a similar orientation of CA and CT on the AgNP surface. Indeed, a different molecular orientation would most probably result in a different spectral shape, at least in terms of the peak relative intensities [73]. Thus, we speculate that the molecule-AgNP interaction takes place via the catechol moieties for both CA and CT (Figure 3b), leaving the carboxylic group of CA on the external part of the molecular layer with respect to the NP surface. In the CA SERS spectrum, additional bands around 1600 cm^−1^ are found. These are also visible in the CA Raman spectrum: by analogy, they can be associated with the carboxylic group. Since the relative intensity of SERS peaks can reflect the local field enhancement, the moderate spectral weight associated with the bands at 1600 cm^−1^ implies that the carboxylic group is located far from the metal surface, where the field amplification is weaker, further supporting our hypothesis of the molecular orientation.

The DFT-calculated SERS spectra for CA and CT are shown in Figure 3c. For CA, the deprotonated form was selected, being the dominant one in neutral conditions. Similarly to the Raman case, the DFT theory predicts the presence of the two main SERS peaks around 1600 cm^−1^. In the low-frequency region, the DFT spectrum reproduces the observed spectral shape quite accurately, predicting, however, an intensity much lower than that observed, particularly for the spectral structures around 1300 cm^−1^. This discrepancy is common to all the theoretical SERS spectra associated with the various CA-Ag conformations considered for the calculations (see Figure S2 in the Supplementary Materials). In this regard, it should be pointed out that, to date, the theoretical estimate of the SERS intensities is still considered challenging, due to the intrinsic difficulty of reproducing this phenomenon within simple DFT [74]. Furthermore, we stress that the experimental SERS spectrum here is collected from dried samples, consisting of multiple molecules assembled on the AgNP surface, while the DFT spectra (both Raman and SERS) are calculated for a single, isolated CA molecule interacting with a single Ag atom in the SERS case. As detailed in Section A of the Supplementary Materials, a growth in the intensity of the spectral structure around 1300 cm^−1^ is observed when the Raman spectrum of CA is collected from dried samples, compared to the spectra of CA in the solution (see Figure S1). The DFT Raman spectrum reproduces the experimental signature of CA in the solution well (that is, in the isolated molecule form), failing to predict the relative intensity observed from the CA powder. This suggests that an enhancement in the band at ∼1300 cm^−1^ can be associated with intermolecular interactions occurring on dried samples. The phenomenon might give rise to the peculiar spectral changes observed both in powder Raman and SERS spectra, explaining as well, in both cases, the discrepancy between the (single molecule) calculations and the (ensemble) experiment.

Despite failing to predict the relative peak intensity, our theoretical calculation can be used to assess the presence and position of the CA SERS active vibrational modes over the whole studied frequency interval, as witnessed by the comparison with the experimental data. For this reason, we carried out a comparison of the experimental spectrum with each of the computed SERS spectra for the different CA-Ag atom configurations used for DFT (Figure S3 of the Supplementary Materials). A very good agreement is observed for the two configurations with the CA-Ag interaction taking place via the CT moiety, further corroborating our hypothesis on the polyphenol orientation on the Ag surface.

In the case of CT, a very good agreement between experimental and theoretical SERS spectra is observed, with DFT accurately predicting the position of the main spectral bands (around 1600 cm${}^{-1}$, in the 1000–1500 cm${}^{-1}$ region, and around 750 cm${}^{-1}$), with minor discrepancies on the peak relative intensities. Thus, the experimental CT spectrum appears to be accurately predicted by the simple theoretical model of a single-molecule-Ag atom interacting system. This means that differently from CA, CT intermolecular interactions do not play a crucial role in determining the overall SERS spectral shape. In particular, both experiments and theory pointed out a significant increase in the intensity of two modes at 1264 and 1552 cm^−1^, which appear rather weak in the CT Raman spectrum.

The well-recognizable and intense SERS response shows that both CA and CT can coordinate the NP surface silver atoms. This is a very important aspect in view of the design of polyphenol-AgNP-based drug delivery applications. Polyphenol-conjugated Hyd@AgNP SERS spectra, together with the DFT spectra and the previously discussed Raman measurements, constitute the reference database for the nano-spectroscopy study of polyphenol-synthesized AgNPs.

### 3.3. Caffeic Acid-Synthesized AgNPs

**Figure 4:**
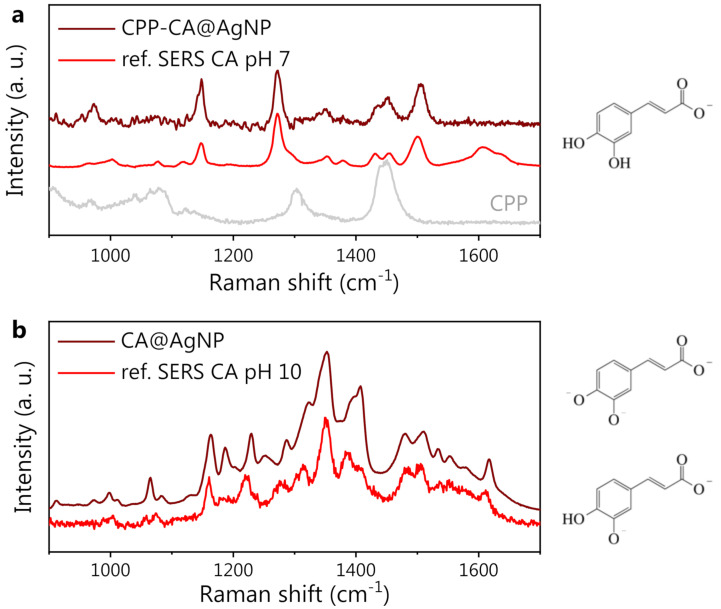
(**a**) From bottom to top: CPP SERS reference spectrum (gray), CA SERS reference spectrum at pH 7 (red), and SERS spectrum of CA-synthesized AgNP in the presence of CPP (CPP-CA@AgNPs, wine). (**b**) From bottom to top: CA SERS reference spectrum at pH 10 (red), and SERS spectrum of CA-synthesized AgNP in the absence of CPP (CA@AgNPs, wine). On the right side of the figure, the CA forms (protonated/deprotonated/doubly deprotonated) expected at neutral (7, top) and highly alkaline (10, bottom) pH are shown.

The SERS spectra of both CPP-CA@AgNPs and CA@AgNPs are shown in Figure 4. The useful reference SERS spectra of CA and CPP are also shown. From the data in panel a, it is evident that the CPP bands are not recognizable in the CPP-CA@AgNPs SERS spectrum. This implies that, as expected from the synthesis, the CPP molecules are rather far from the NP surface, not benefiting from the local field enhancement. The remarkable resemblance between the CPP-CA@AgNP spectrum and the reference CA SERS spectrum at neutral pH is further proof of this claim. The lower intensity of the bands associated with the carboxyl group, around 1600 cm^−1^, suggests a lower SERS enhancement compared to that of the reference SERS measurements, possibly associated with a different NP packing, increasing the interparticle distance in the SERS hotspots. Less-efficient SERS hotspots decrease the visibility of SERS bands, particularly of those associated with functional groups located far from the NP surface. For a detailed discussion on the available literature data on CA SERS spectra, please refer to Section B in the Supplementary Materials.

As evident from Figure 4b, the CA@AgNP SERS spectrum appears completely different from the CPP-CA@AgNP one. The characteristic CA peaks in Figure 4a appear shifted (see, e.g., the band at 1145 cm^−1^ in panel a, which is blue-shifted by about 20 cm^−1^ in panel b) and, most importantly, the appearance of a broad and intense spectral structure can be observed, with peaks at 1350 and 1400 cm^−1^. Considering the pH-dependent modifications of CA and the high pH value used for the CA@AgNPs sample preparation, appropriate reference SERS measurements on CA at increasing pH were acquired, using Hyd@AgNPs as SERS substrate. The spectra at pH >10 appeared rather noisy and could not be used as a SERS reference. Nevertheless, a very good agreement between the CA reference SERS spectrum collected at pH 10 and the CA@AgNP (pH 12) SERS spectrum is observed. The spectral changes observed on CA at high pH can be ascribed to the chemical changes induced by the second and third deprotonations of CA molecules and to a possible associated reorientation on the AgNP surface, as previously observed with other pH-responsive analytes [73].

**Figure 5:**
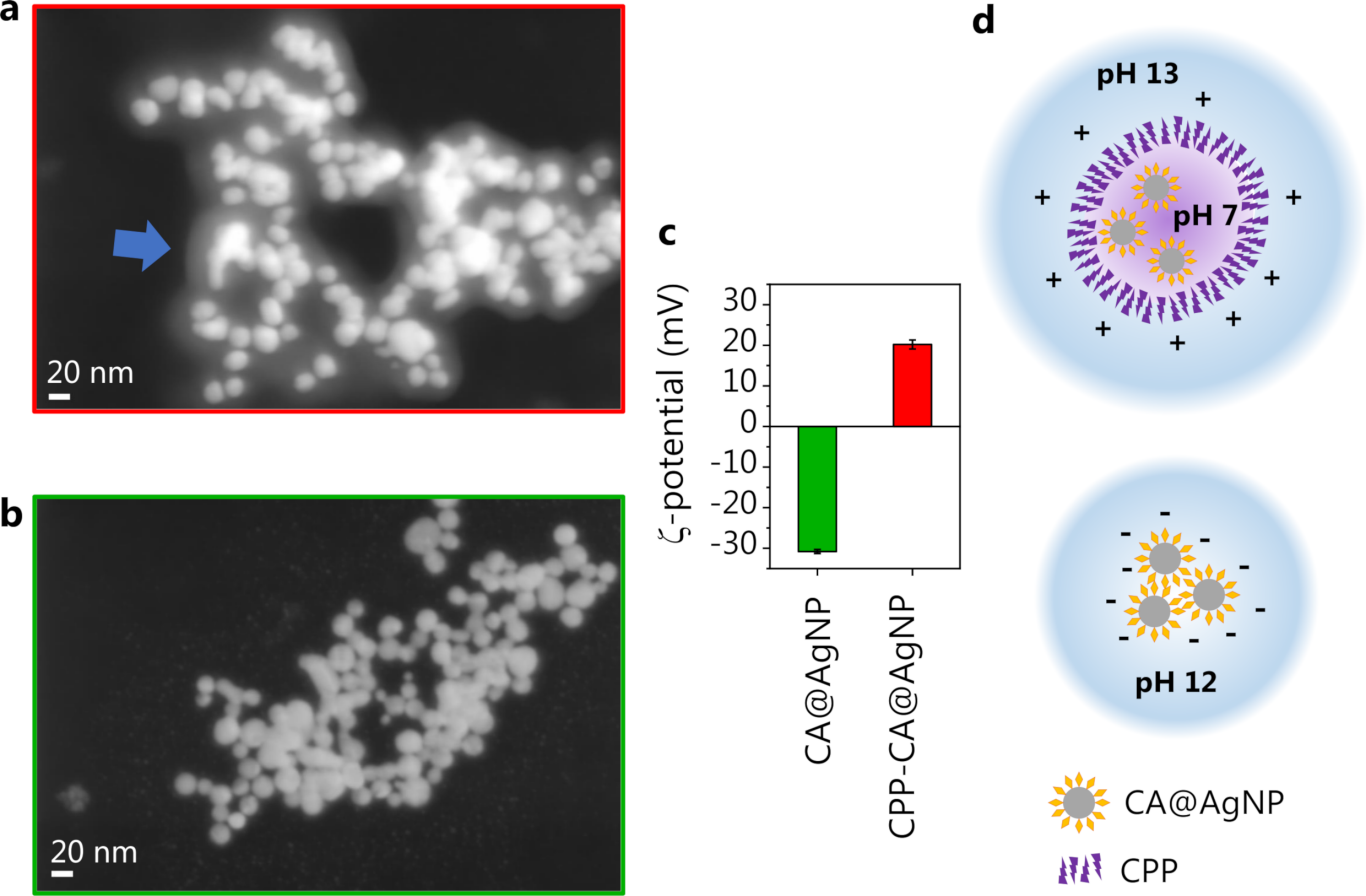
(**a**) CPP-CA@AgNP SEM image. The arrow highlights the observed CPP layer. (**b**) CA@AgNP SEM image. (**c**) ζ-potential measurement on the two nanosystems. (**d**) Sketch of the constituted nanosystems.

From these observations, it is clear that the role of CPP in the CPP-CA@AgNP system is to screen the CA molecules anchored on the silver surface, protecting them from the extreme environmental pH condition and preventing their deprotonation even at the extremely high pH values of the solution (pH 13). This is coherent with the observation reported in [75] that the CPP acts as a stabilizer for the polyphenol-synthesized AgNPs, by the formation of an external corona. This leads to the observation, on the CPP-CA@AgNPs, of a spectral shape similar to that observed on reference CA spectra at neutral pH (Figure 3). This hypothesis is supported by the SEM images of CPP-CA@AgNPs and CA@AgNPs (Figure 5a,b, respectively). Indeed, the CPP-CA@AgNPs seem to be enveloped by a layer of poorly scattering organic material, which does not appear in the CA@AgNP sample and can thus be ascribed to the aforementioned CPP corona [75]. This is confirmed by the light transmission spectroscopy (LTS) measurements and by the data analysis based on the Mie scattering theory, presented in Section E of the Supplementary Materials [35]. The estimates of the hydrodynamic diameters of the colloidal systems obtained by dynamic light scattering (DLS) corroborated the results obtained by LTS (Supplementary Materials, Section E). The much wider dispersion in size estimated by LTS as well as DLS for the CPP-containing sample is reasonably associated with the presence of the CPP corona. The different sign of the ζ-potential measured for the two samples (Figure 5c), associated with the opposite sign of the surface charge, is further proof in favor of the presented description of the nanosystem (see the sketch in Figure 5d). Indeed, on CA@AgNPs, the surface charge is negative, as expected from the SERS observations suggesting the molecular deprotonation on the NP surface. On the other hand, the surface charge of CPP-CA@AgNPs is positive because of the presence of the cetyltrimethylammonium chloride CPP. The absolute value of the ζ-potential, between 20 and 30 mV, points out a moderately stable colloidal dispersion [76].

On the CA@AgNP sample, we observed a significantly higher overall SERS intensity compared to CPP-CA@AgNPs (detailed discussion in Section D of Supplementary Materials). This can be attributed to several concurring effects. The first is the steric hindrance of the CPP layer: when the AgNP dispersion is dried on a solid support for SERS measurements, the CPP molecules keep the NPs rather far apart and hinder the formation of efficient hotspots by preventing the closely packed aggregation of NPs. This is evident in the SEM images (Figure 5a,b) from both the average interparticle distance (larger for CPP-CA@AgNPs) and the number of hotspots formed in an aggregate (lower for CPP-CA@AgNPs), decreasing the SERS efficiency in the CPP-containing sample. Another important aspect to consider is the deprotonation level of the CA molecule in the CPP-free sample. The concentration of synthesized NPs assessed by LTS (Section E of the Supplementary Materials), which is higher by an order of magnitude in the CPP-free sample, suggests that the yield in NP formation increases when the CA is deprotonated because of the extremely alkaline (pH 12) reaction mix, coherently with previous observations [23]. This results in a higher SERS response retrieved in the CA@AgNP sample. Furthermore, the polarizability of CA, when subjected to multiple deprotonations, might increase, as pointed out as well by DFT calculations (data not shown), thus increasing the CA SERS cross-section in the CPP-free sample.

## 4. Conclusions

In this study, the nanoscale spectroscopic investigation of AgNPs synthesized using phenolic compounds as reducing agents was carried out. The high specificity and sensitivity of SERS were exploited with the aim of assessing the resulting surface chemistry as a basis for biomedical perspective applications. The ab initio DFT calculations of the SERS spectra of the Ag-conjugated phenolic compounds were successfully used to assign the SERS peaks to well-identified vibrational modes and to interpret how molecular modifications affect the SERS fingerprint. It is worth stressing that, due to the chemical susceptibility of polyphenolic compounds, the identification of their SERS spectral signature is rather elusive. The approach proposed here, composed of independent experimental and theoretical reference investigations, suggests a route that can be successfully applied to the study of other responsive polyphenolic-AgNP compounds and that can allow access to the chemical modifications of the polyphenol species occurring upon a changing environment. In detail, we here demonstrated that the CA deprotonation degree can be controlled and varied depending on the presence of CPP, suggesting an experimental tuning, by synthesis, of the interaction of the CA with the NP surface. Furthermore, by combining optical spectroscopy and electron microscopy methods, we demonstrated that the CA deprotonation degree impacts the NP characteristics and the synthesis reaction yield. In perspective, the proposed pH-dependent DFT and SERS analysis can be extended to more complex polyphenols already employed for pharmaceutical applications, including catechin, epigallocatechin gallate, and others.

The pH responsivity of SERS-active nanosystems is a property that is readily exploited for nanoscale pH sensing in diverse biological environments [77], e.g., the acidic extracellular environment of cancer cells [73]. Here, the pH-responsivity of CA in the 4–10 pH range (and possibly even further) is demonstrated, suggesting the potential perspective applicability of the present nanosystem as a traceable and biocompatible SERS pH nanosensor. Among the biological environments where such a pH measurement might be useful, one can mention the quantification of the internal pH of lysosomes or other acidic organelles, where the pH gradients reach values in the 3.8–6.6 range, i.e., over the first pK*_a_* of CA [78]. Furthermore, this investigation is promising to design controlled, chemically active nanovectors to be employed as drug nanocarriers for in vitro and in vivo applications. In particular, the proposed study offers a strategy that allows: (i) predicting the available and active functional groups onto the AgNPs, able to work as anchoring sites for chromophores, bio-tags, drugs, etc.; (ii) hypothesizing and designing ‘ad hoc’ release mechanisms for drugs in correspondence to biological targets, as receptors or cells, possibly exploiting the pH responsivity of the nanosystem [79]. For example, the gradual protonation of CA in the presence of acidic environments, and the consequent change in the surface charge of the nanocarrier, might trigger the pH-dependent release of a specific drug anchored on the nanocarrier by electrostatic interaction [80]. Thus, the results of this work pave the way for the further functionalization of polyphenol-AgNP biomaterials, potentially exploiting both the polyphenol binding affinity to important biological molecules and its pH responsivity, toward effective biomedical applications.

## Data Availability

The data presented in this study are available from the corresponding authors upon reasonable request.

## Supplementary Materials

The following Supplementary Materials can be downloaded at: https://www.mdpi.com/article/10.3390/nano13030377/s1; Figure S1: caffeic acid Raman spectra in methanol and water solution, and in the powder form; Figures S2 and S3: theoretical SERS spectra for different caffeic acid - Ag atom three-dimensional configurations; Figure S4: theoretical SERS spectra for different catechol - Ag atom three-dimensional configurations; Figure S5: comparison between SERS and UV-Visible spectra of caffeic acid synthesized AgNPs with and without the capping agent; Figures S6–S8: particle size distribution for the two aforementioned systems obtained by SEM, LTS and DLS, respectively. Data are discussed by making use of references [35,76,81,82,83,84,85,86,87,88,89,90,91].
