# Supplementary material for: Nanoscale Surface-Enhanced Raman Spectroscopy Investigation of a Polyphenol-Based Plasmonic Nanovector
Source: Nanomaterials (Basel). 2023 Jan 17;13(3):377. doi: 10.3390/nano13030377 (PMC9921702; doi:10.3390/nano13030377)
Supplement: Supplementary file 1 [file nanomaterials-13-00377-s001.zip › supplmat.pdf]

Supplementary Materials: Nanoscale Surface-Enhanced Raman Spectroscopy Investigation of a Polyphenol-Based Plasmonic Nanovector

Giacomo Nisini, Annalisa Scroccarello, Francesca Ripanti *, Claudia Fasolato *, Francesco Cappelluti, Angela Capocefalo, Flavio Della Pelle, Dario Compagnone and Paolo Postorino

Section A. Raman spectrum of caffeic acid: bulk vs solution.

The Raman spectrum of bulk (powder) caffeic acid (CA) shows considerable differences compared to the spectrum of CA in solution. These differences are relevant for the interpretation of the surface enhanced Raman scattering (SERS) spectra of CA, presented in the main text. In Figure S1, the spectra of CA in water and methanol solution and that of powder CA are displayed. A shift and change in the relative intensity of the vibrational bands is appreciable in comparing the solution spectra to the solid state spectrum. In particular, an enhancement of the spectral structure around 1300 cm^{-1} (bands at 1286 cm^{-1}, 1299 cm^{-1}, and 1305 cm^{-1}) is observed in the CA powder spectrum compared to the solution spectra, where only weaker peaks appear at 1272 cm^{-1} and 1287 cm^{-1}. On the opposite, the CA powder and solution spectra appear rather similar in the [1450 - 1750] cm^{-1} range, the main appreciable difference being the increased width of Raman peaks in solution, as commonly observed for other molecules.

The differences between the powder and solution Raman spectra can be ascribed to two main effects. The increase in the intensity of some peaks in the bulk spectrum might derive from the (almost) crystalline CA state in the powder sample: some peaks, considerably weak in solution, might appear enhanced in the solid CA spectrum due to the interactions between adjacent, closely packed CA molecules. On the other hand, for solution spectra, an important aspect is the molecule-solvent interactions, producing an increase of the width and a shift of the spectral bands, and possibly a further change in the relative spectral intensities. The considerations on the CA solutions hold for both water and methanol, where the polyphenol is highly soluble.

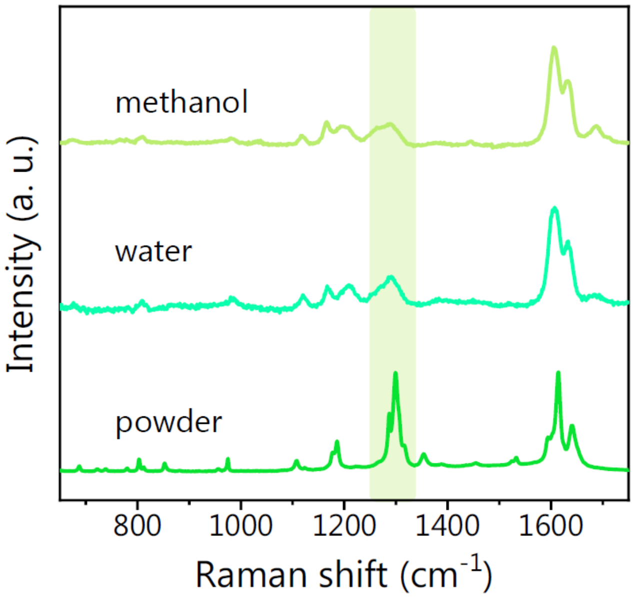

Figure S1. From bottom to top: CA powder Raman spectrum (green), Raman spectrum of CA in water (cyan), Raman spectrum of CA in methanol (light green). The frequency region shadowed in gray highlights the spectral structure around 1300 cm^{-1} characterized by a marked intensity variation.

Section B. Further discussion on reference polyphenol SERS spectra.

In Figure 3 of main text we report a comparison between the Ag colloid (hydroxilamine-synthesized silver nanoparticles, Hyd@AgNPs) blank spectrum and the SERS spectra collected after the conjugation of Hyd@AgNPs with polyphenols. The appearance of strong vibrational peaks between 1000 cm^{-1} and 1600 cm^{-1} in the latter is a marker of the polyphenols in proximity to the AgNP surface. As mentioned in the main text, an almost complete overlapping between CA and catechol (CT) SERS spectra is observed. We associate it to the resemblance between the CA and CT chemical structure. This implies that the main contribution to the total SERS intensity comes from the CT moiety, which is a subunit of the CA chemical structure. The only relevant difference between CA and CT SERS spectra is the different intensity of the vibrational bands at $\sim$ 1610 cm^{-1}, which appear much lower in the CT spectrum. According to the band assignment for CA, these vibrational modes are ascribed to a composition of C=C and C=O stretching modes. The observed lack of intensity in the CT spectrum is thus due to the absence of a carbonyl moiety.

In the scientific literature, only a few experimental reports are available on the SERS response of polyphenols. Among these, the work of S. Sánchez-Cortés, J. V. García-Ramos et al. is worth mentioning. They carried out extensive studies where they analyzed by SERS the polymerization of some polyphenol species, including CA and CT, depending on different parameters, such as solvent, pH, excitation wavelength, exposure to UV-Vis light, and presence of AgNPs [1–3]. They demonstrated that the SERS signature of polyphenols reflects the subtle molecular modifications occurring upon chemical reactions. Due to their marked reactivity, polyphenols are easily involved in chemical reactions, which makes their SERS signature somewhat elusive. The spectral features Sanchez-Cortes et al. observe are similar, although not completely overlapping, with those we presented in the main text.

Indeed, our SERS measurements were collected on freshly prepared samples, avoiding UV exposure, using a long (633 nm) excitation wavelength and keeping a low power density (about 5 kW/cm^2), well below the threshold signal for many well-known plasmon-induced chemical reactions [4]. This experimental design allowed us observe a stable spectral shape, characteristic of each sample, in our SERS experiments. Based on the strong specificity of the SERS molecular fingerprint, and the absence of variations in the SERS spectral shape, one can state that the employed conditions prevented the formation of chemical products derived from CA reactions, neither triggered by high energy photons or high laser powers, which were not delivered to the samples, nor involving CA molecules and Ag surface atoms as catalyzers. Concerning the second class of reactions, the observed similarity between the reference SERS spectra of CA, obtained using Hyd@AgNPs as SERS substrate, and that of the (CPP-)CA@AgNPs, can be considered a further proof of the absence of such reactions. Indeed, in the reference experiments, exploiting Hyd-conjugated AgNPs, the CA molecules did not encounter a free Ag surface to react with: the high (negative) surface charge of Hyd@AgNPs synthesized with the method here adopted [5] demonstrates the presence of Hyd molecules on the metal surface. Thus, the catalytic action of the Ag surface for Hyd@AgNPs would be hindered compared to the CA@AgNP case. The fact that the observed SERS spectral shape is clearly overlapping in the reference measurements and in measuring the CA@AgNP and CPP-CA@AgNP samples, where the CA molecules are adjacent to the Ag surface, bring us to the reasonable conclusion that the same molecular species are observed in both experiments, and that the main spectral variations observed (panels a vs b in Figure 4, main text) can be explained in terms of the environmental pH, as discussed in the main text. The oxidation process occurring during the synthesis of (CPP-)CA@AgNP, responsible for the formation of NPs, does not seem to affect the overall SERS spectrum, which is identical to the reference spectrum. These considerations bring us to the conclusion that our protocol was effective in avoiding the formation of chemical species different from CA.

Our spectra are in agreement with those reported in two more recent studies. In the first one, the authors performed a systematic SERS analysis of different polyphenols,

including CA [6]. They used silver colloids as SERS-active materials and collected the SERS signal as a function of the polyphenol concentration. In the latter, a titanium-oxide cluster was exploited as SERS substrate for phenol and CT detection [7]. None of these studies, however, presents a systematic comparison of SERS spectra from different metal substrates and at different pH conditions, nor a theoretical analysis. Here, we expand this body of studies using SERS as a tool for molecular investigation at the nanoscale.

Section C. Theoretical SERS spectra: effects of polyphenol-Ag conjugation.

In Figure S2 we show the theoretical CA SERS spectra for five different configurations of the CA-Ag system, along with the average CA theoretical SERS signature, computed as the weighted average of the five conformer spectra. As detailed in the Materials and Methods section, the interaction of CA with the Ag surface is accounted for by including an Ag atom in the calculation, located in different positions with respect to the molecule.

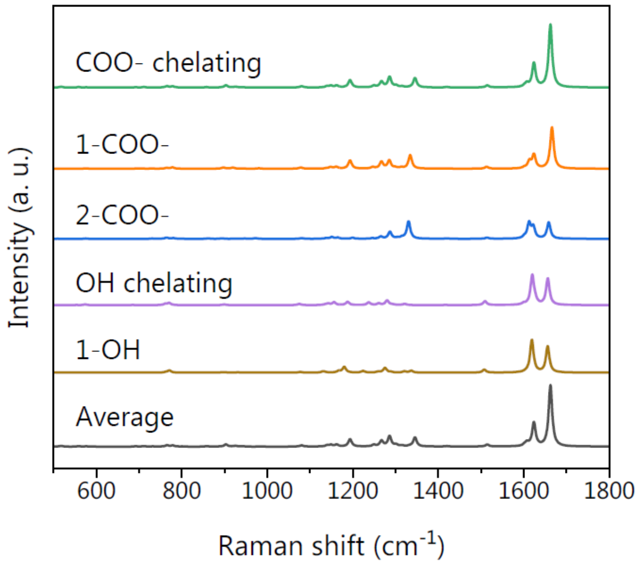

Figure S2. CA theoretical SERS spectra for different interacting configurations of the molecule and Ag atom. From the bottom to the top: CA average SERS spectrum, 1-OH configuration, OH chelating configuration, 2-COO^- configuration, 1-COO^- configuration, COO^- chelating configuration.

As expected [8,9], the number and frequency of the main SERS bands is similar in all the computed spectra, while the relative intensities, and thus the spectral shape, strongly depend on the part of the molecule interacting with the Ag atom. Indeed, even if our theoretical model does not include the field enhancement due to the electromagnetic SERS mechanism (EEM), the interaction of the CA molecule with the silver atom causes significant changes to the polarizability, and hence to the Raman cross section of specific vibrational modes, depending on the particular CA-Ag configuration as predicted by the calculations. In this regard, the proposed theoretical model can be thought as a first approximation of the chemical enhancement mechanism (CEM).

In the experimental spectrum (Figure 3 in the main text) we noticed that the SERS enhancement of vibrational bands mainly affects the [1000-1550] cm^{-1} region. In the computed spectra of Figure S2, the same frequency region is not characterized by intense vibrational peaks, associated to the missing EEM in our theoretical model. On the other hand, in the experimental spectrum we did not observe remarkable SERS-induced modifications in the CA spectral shape around $\sim$ 1600 cm^{-1}. With this in mind, we performed a systematic comparison, in the [1550-1750] cm^{-1} region, between each conformer theoretical spectrum and the experimental one (Figure S3). We noticed that the relative intensity of the two peaks at 1600 cm^{-1} and 1612 cm^{-1}, for the three configurations with the Ag atom adjacent to the COO^- group (Figure S3a, b and c), does not reproduce the experimental

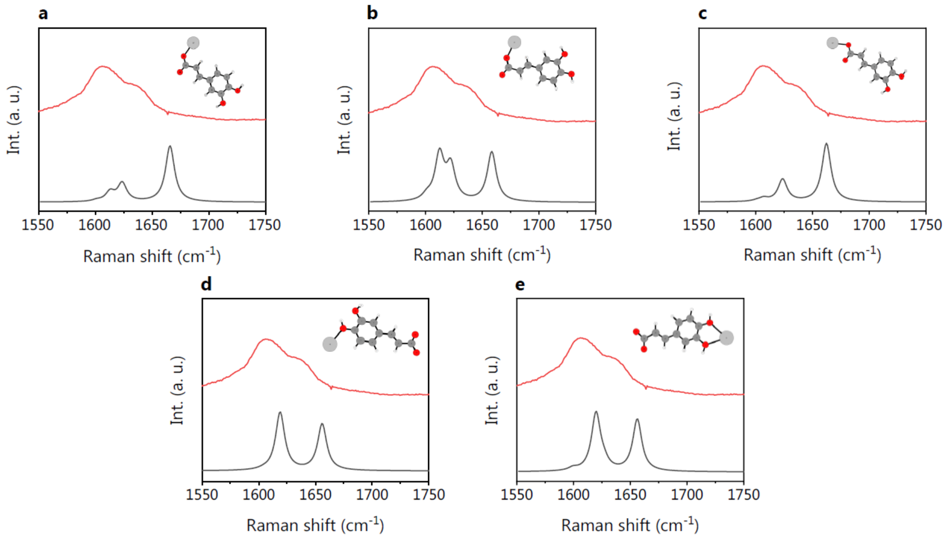

Figure S3. Comparison between the experimental SERS spectrum (red) of CA and theoretical SERS spectra (black) of different CA-Ag configurations in the [1550 and 1750] cm^{-1} region. (a) 1-COO^- configuration. (b) 2-COO^- configuration. (c) COO^- chelating configuration. (d) 1-OH configuration. (e) OH chelating configuration.

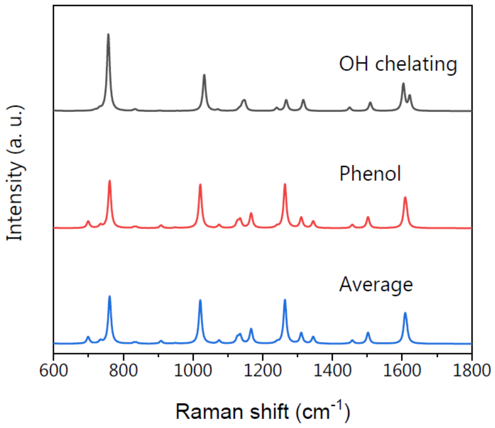

Figure S4. CT theoretical SERS spectra for different interacting configurations between molecule and silver atom. From the bottom to the top: average weighted SERS spectrum, Phenol configuration, OH chelating configuration.

observations. On the contrary, the same intensity ratio for the 1-OH and the OH chelating configurations (Figure S3d and e) is in good agreement with the experimental observations. The latter configurations can thus be regarded as the most probable.

The theoretical results provide some information on the interaction between CA and AgNPs. Indeed, as discussed in the main text, considering the resemblance between the experimental CA and CT SERS spectra, and their similar chemical structure, we hypothesize that the interaction between CA and the AgNP surface occurs *via* the oxygen atoms of the catechol moiety. The same molecular orientation with respect to the silver surface might occur in the CT case, thus explaining the observed similarities. In Figure S4, we report the theoretical CT SERS spectra for two different CT-Ag system configurations along with the CT average SERS spectrum.

Section D. Effect of the capping agent on the SERS response of CA@AgNPs

In Figure S5a, a comparison between the spectra of caffeic acid-synthesized AgNPs in absence (CA@AgNPs) and in presence of the cetyltrimethylammonium chloride capping

layer (CPP-CA@AgNPs SERS) is displayed. The same spectra are shown in the main text with a different normalization. Figure 4 of the main text is indeed intended to emphasize the difference between spectral shapes, in terms of number and position of the observed vibrational bands, hence the spectra are normalized to the most intense peak for each nanosystem. Here, instead, spectra are normalized to the common AgNP peak around 230 cm^{-1}, allowing for a direct comparison between the overall spectral intensities. It is evident that the CA@AgNPs SERS spectrum is much more intense than the CPP-CA@AgNPs one, resulting in an overall enhancement of two orders of magnitude of the fingerprint SERS spectrum of CA in the CPP-free sample. This drastic difference between the observed intensities is motivated by several concurring effects, as detailed in the following.

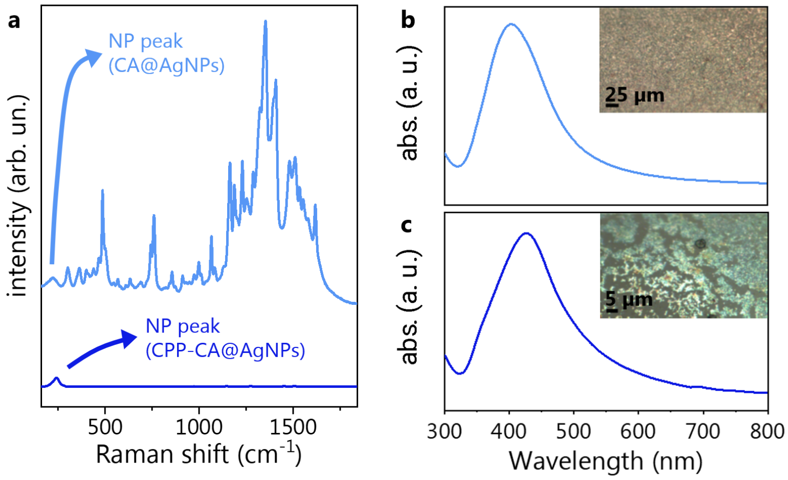

Figure S5. (a) comparison between CPP-CA@AgNPs (blue) and CA@AgNPs (cyan) SERS spectra. Spectra are normalized to the AgNP peak at ∼ 230 cm^{-1}. UV-Vis absorption spectrum of CA@AgNPs (b) and CPP-CA@AgNPs (c) water dispersion. In the insets, microscopic images of the samples (100x objective) obtained by dropcasting the samples on a glass slide and drying at ambient conditions.

First of all, as discussed in the main text, the change in the SERS intensity is accompanied by a remarkable change in the SERS spectral shape, which is associated to the deprotonation of the CA molecule, occurring at alcaline pH and recorded on the CPP-free CA@AgNP sample. In commenting Figure 4, we suggested that the CPP molecules act as a shield for the NP bonded CA molecules, screening the high pH of the solution. On the opposite, when no CPP is available, the NP bonded CA molecules are exposed to an extremely alcaline pH, causing multiple CA deprotonations. The deprotonated CA molecules might exhibit a different affinity for the Ag surface, resulting in a higher molecular density on the NP surface or, more probably, the electronic polarizability of deprotonated CA might result in a higher Raman cross section, hence leading to a higher SERS intensity.

Furthermore, one might hypothesize that the CA reducing power is enhanced when the CA is deprotonated because of the absence of the CPP. This would cause a higher yield in terms of NP formation, as also demonstrated by the analysis of the sample optical density (see Section E). The higher number of NPs produces a larger number of hot-spots, as well visible in the CA@AgNPs and CPP-CA@AgNPs microscopic images, in the insets of Figure S5b and c, respectively. The much higher density of hot-spots results in a higher SERS intensity.

Another crucial aspect in the determination of SERS intensity is the interparticle distance within hot-spots. In the case of CPP-CA@AgNPs, the steric hindrance of CPP increases the interparticle distance while decreasing the packing density of the NP aggregates, as well visible from the SEM images of the samples in Figure 5 of the main text. This implies a lower SERS efficiency of the CPP-CA@AgNP sample.

Section E. Nanoparticle formation by caffeic acid: size, reaction yield and effect of surfactant.

The CA@AgNPs and CPP-CA@AgNPs colloids were characterized by Scanning Electron Microscopy (SEM), Dynamic Light Scattering (DLS) and Light Transmission Spectroscopy (LTS).

AgNp size from the statistical analysis of SEM images

SEM images (Figure 5 in the main text, and others) have been analyzed by employing Gwyddion 2.56 free software. For each sample, 80 horizontal profiles have been extracted from the images. The obtained particle size distribution are reported in Figure S6. The average particle size obtained by fitting data to a lognormal distribution is 23.3 ± 5.6 nm for CA@AgNP and 22.4 ± 5.5 nm for CPP-CA@AgNPs.

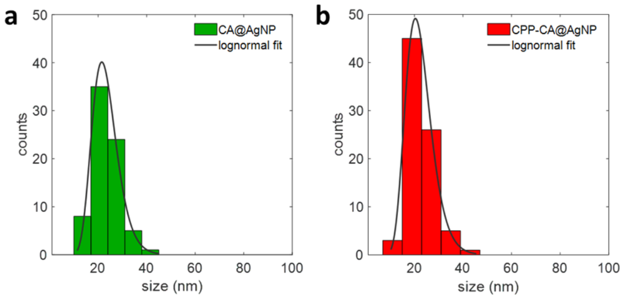

Figure S6. Particle size distribution obtained by the analysis of SEM images for CA@AgNP (a) and CPP-CA@AgNP (b). The black curve indicates the fitting to a lognormal distribution.

Size and concentration of AgNPs by LTS

An estimate of the effective size and concentration of CA@AgNPs and CPP-CA@AgNPs in the colloidal form is obtained by Light Transmission Spectroscopy (LTS). The extinction spectrum of the samples was measured in a wide wavelength range between 190 and 1300 nm by employing a double ray spectrophotometer (v-590, Jasco) and quartz cuvettes with 1 mm path length.

The extinction spectra corresponding to CPP-CA@AgNPs and CA@AgNPs samples are reported in Figure S7a and b, respectively. The extinction spectra are inverted by using a mean square root-based algorithm that gives the particle density distribution as a function of their radius through the Beer-Lambert law and the Mie scattering cross section of the particles, modeled as spheres [10,11]. The complex refractive of silver by Johnson and Christy [12] was employed in all calculations.

This technique allows us to estimate with a single measurement the average radius of the AgNPs in solution and the total number of particles per milliliter, which is calculated from the integral of the particle density distribution. The LTS analysis performed on the CPP-CA@AgNPs and CA@AgNPs samples is reported in Figure S7c and d.

The estimated particle diameters are (72.8 ± 26.8) nm for CPP-CA@AgNPs and (78.4 ± 4.8) nm for CA@AgNPs. Both the obtained values result higher with respect to the particle size measured by SEM. This can be explained with a weak effect of aggregation of the colloidal particles in solution. This partial aggregation of the AgNPs is consistent with the measured ζ-potential (Figure 5 in the main text), whose absolute values are well below 40 mV, pointing out a moderately stable colloidal dispersion [13]. The much larger dispersion of the CPP-containing sample can be associated to the effect of the CPP corona, discussed in the main text. Concerning the absolute concentration of particles in solution, we measure $1.50 \cdot 10^{13}$ part/mL for CPP-CA@AgNPs and $1.52 \cdot 10^{14}$ part/mL for CA@AgNPs. The higher number of particles in solution in the case of CA@AgNPs points out to a better yield of the synthesis process, probably associated to the CA deprotonation.

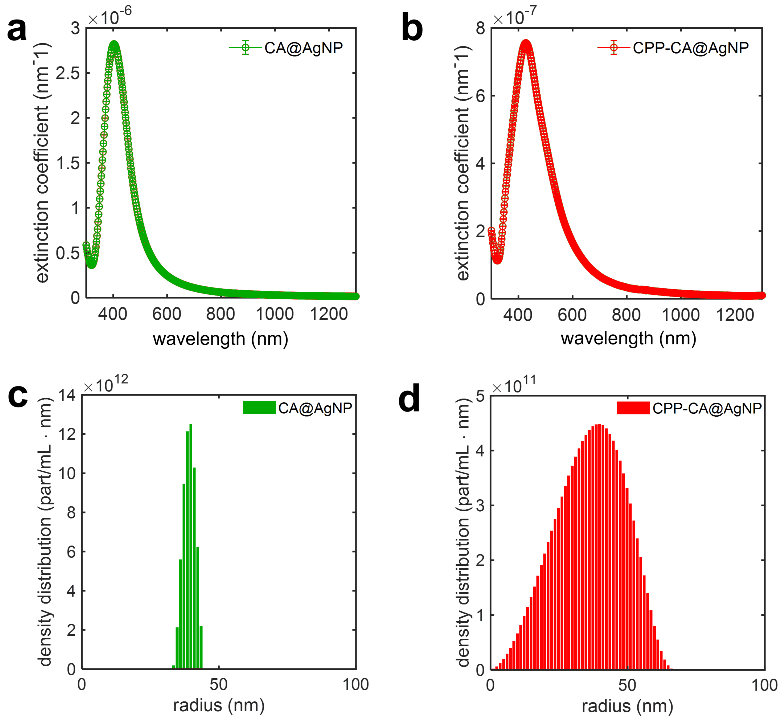

Figure S7. LTS analysis on the two CA-synthesized AgNP samples. Top row: extinction spectra of CPP-CA@AgNPs (a) and CA@AgNPs (b). Bottom row: particle density distributions obtained by Mie inversion algorithm for CPP-CA@AgNPs (c) and CA@AgNPs (d).

Hydrodynamic diameter from DLS measurements

The average hydrodynamic diameter of the silver colloids in solution was obtained by DLS employing the same NanoZetaSizer apparatus used for ζ-potential measurements. Briefly, the intensity autocorrelation functions were acquired at an angle of 173° and correlograms were analyzed with the CONTIN algorithm to extrapolate the associated decay times. The decay times were used to determine the distribution of the diffusion coefficients D of the particles, which in turn were converted to a distribution of hydrodynamic diameters $2R_H$ using the Stokes-Einstein relationship $R_H = \frac{k_B T}{6\pi\eta D}$ where $k_B T$ is the thermal energy and η the solvent viscosity. The obtained intensity weighted distributions of the hydrodynamic diameter are reported in Figure Y.

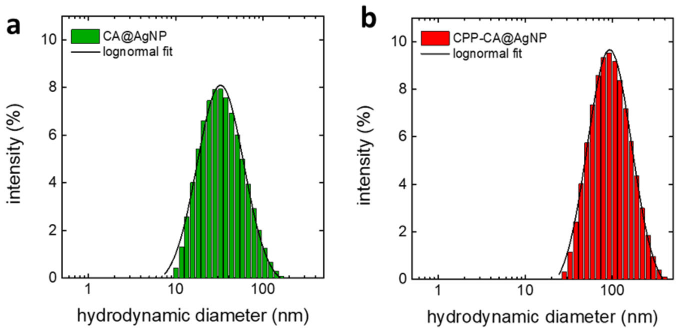

Figure S8. Intensity weighted distribution of the hydrodynamic diameter obtained by DLS for CA@AgNP (a) and CPP-CA@AgNP (b). The black curve indicates the fitting to a lognormal distribution.

The center value of the distributions were determined by fitting the data to a lognormal distribution, obtaining a hydrodynamic diameter of 57.9 ± 39.7 nm for CA@AgNP and of 154.3 ± 97.6 nm for CPP-CA@AgNP. Considering the large uncertainties, the values of the estimated hydrodynamic diameters are fully compatible with the size obtained by LTS. In the case of CPP-CA@AgNPs, the estimated hydrodynamic diameter (DLS) is larger than the aggregate effective size: we interpret this discrepancy in terms of the interaction of the CPP surfactant molecules with the solvent, which might increase the hydrodynamic diameter in the CPP-CA@AgNP case compared to the CA@AgNP system.

References

1. Sánchez-Cortés, S.; Garcıa-Ramos, J. Photoinduced coupling and adsorption of caffeic acid on silver surface studied by surface-enhanced Raman spectroscopy. *Spectrochimica Acta Part A: Molecular and Biomolecular Spectroscopy* **1999**, *55*, 2935–2941.
2. Sánchez-Cortés, S.; García-Ramos, J. FT surface-enhanced Raman evidence of the oxidative condensation reactions of caffeic acid in solution and on silver surface. *Applied Spectroscopy* **2000**, *54*, 230–238.
3. Sanchez-Cortes, S.; Francioso, O.; Garcıa-Ramos, J.; Ciavatta, C.; Gessa, C. Catechol polymerization in the presence of silver surface. *Colloids and Surfaces A: Physicochemical and Engineering Aspects* **2001**, *176*, 177–184.
4. Zhang, Z.; Kneipp, J. Surface Molecular Patterning by Plasmon-Catalyzed Reactions. *ACS Applied Materials & Interfaces* **2021**, *13*, 43708–43714.
5. Stewart, A.; Murray, S.; Bell, S. Simple preparation of positively charged silver nanoparticles for detection of anions by surface-enhanced Raman spectroscopy. *Analyst* **2015**, *140*, 2988–2994.
6. Aguilar-Hernández, I.; Afseth, N.K.; López-Luke, T.; Contreras-Torres, F.F.; Wold, J.P.; Ornelas-Soto, N. Surface enhanced Raman spectroscopy of phenolic antioxidants: a systematic evaluation of ferulic acid, p-coumaric acid, caffeic acid and sinapic acid. *Vibrational Spectroscopy* **2017**, *89*, 113–122.
7. Liu, C.; Hu, J.; Biswas, S.; Zhu, F.; Zhan, J.; Wang, G.; Tung, C.H.; Wang, Y. Surface-enhanced Raman scattering of phenols and catechols by a molecular analogue of titanium dioxide. *Analytical Chemistry* **2020**, *92*, 5929–5936.
8. Cabalo, J.; Guicheteau, J.A.; Christesen, S. Toward understanding the influence of intermolecular interactions and molecular orientation on the chemical enhancement of SERS. *The Journal of Physical Chemistry A* **2013**, *117*, 9028–9038.
9. Marshall, A.R.; Stokes, J.; Viscomi, F.N.; Proctor, J.E.; Gierschner, J.; Bouillard, J.S.G.; Adawi, A.M. Determining molecular orientation via single molecule SERS in a plasmonic nano-gap. *Nanoscale* **2017**, *9*, 17415–17421.
10. Weese, J. A reliable and fast method for the solution of Fredhol integral equations of the first kind based on Tikhonov regularization. *Computer Physics Communications* **1992**, *69*, 99–111.
11. Sarra, A.; Stanchieri, G.D.P.; De Marcellis, A.; Bordi, F.; Postorino, P.; Palange, E. Laser transmission spectroscopy based on tunable-gain dual-channel dual-phase LIA for biological nanoparticles characterization. *IEEE Transactions on Biomedical Circuits and Systems* **2021**, *15*, 177–187.
12. Johnson, P.B.; Christy, R.W. Optical constants of the noble metals. *Physical Review B* **1972**, *6*, 4370.
13. Sharaf, O.Z.; Taylor, R.A.; Abu-Nada, E. On the colloidal and chemical stability of solar nanofluids: from nanoscale interactions to recent advances. *Physics Reports* **2020**, *867*, 1–84.
